# Update on GH therapy in adults

**DOI:** 10.12688/f1000research.12057.1

**Published:** 2017-11-16

**Authors:** Cesar Luiz Boguszewski

**Affiliations:** 1 Endocrine Division (SEMPR), Department of Internal Medicine, Federal University of Parana, Curitiba, Brazil

**Keywords:** growth hormone, adults with GH deficiency, IGF-I

## Abstract

Over the last three decades, short- and long-term observational studies, clinical trials, systematic reviews, and meta-analyses have provided relevant information on the efficacy and safety of growth hormone (GH) replacement therapy in adults with GH deficiency (AGHD). The knowledge acquired during this time has been compiled into different guidelines that offer clinicians an evidence-based, practical approach for the management of AGHD. There are, however, still open questions in some key areas in which recommendations are supported by only moderate or weak evidence. In the last recent years, the development of long-acting GH preparations has created new therapeutic possibilities by decreasing injection frequency, improving adherence and thereby potentially maximizing clinical outcomes. The aims of this review are to advance our understanding on the diagnosis and treatment of AGHD and to present an update and future perspectives on the use of long-acting GH preparations.

## Introduction

In 1962, a 35-year-old woman with hypopituitarism was the first adult treated with pituitary growth hormone (GH). She felt improvement in physical vigor and psychological well-being after two months of treatment ^1^. At that time, however, limited availability of pituitary GH restricted its therapeutic use to those few children with more severe forms of growth retardation. This problem was solved in 1985 with the introduction in the clinical practice of unrestricted amounts of recombinant human GH, which also created several novel therapeutic opportunities.

It was in this context that a new entity in hypopituitary patients receiving replacement therapy for thyroid-stimulating hormone (TSH), follicle-stimulating hormone/luteinizing hormone (FSH/LH) and/or adrenocorticotropic hormone (ACTH) deficiencies, but not for GH deficiency (GHD), started to be defined as: “adult GH deficiency” (AGHD) ^2^. The initial characterization of AGHD occurred in parallel with a Swedish epidemiologic study that demonstrated an increased mortality in patients with hypopituitarism, mainly related to cardiovascular complications ^3^. This pioneer study was followed by others confirming this finding, and a recent meta-analysis concluded that the risk of premature mortality in hypopituitarism is particularly high in women and in patients diagnosed at a younger age ^4– 7^. However, it is not defined to what extent untreated AGHD is responsible for the reduced life expectancy observed in hypopituitary patients, as many other factors—such as previous exposure to radiation, the etiology of the underlying hypothalamic-pituitary disease, or mistreatment of other associated pituitary hormone deficiencies—might also contribute to increased mortality ^6– 9^. Evidence from a Brazilian kindred with lifetime isolated GHD due to a homozygous mutation in the GH-releasing hormone (GHRH) receptor gene does not support a deleterious effect of congenital isolated GHD on survival ^8^. In this kindred, affected individuals exhibit normal longevity despite their dwarfism, visceral obesity, and the presence of some cardiovascular risk factors. Remarkably, these patients maintain increased insulin sensitivity and do not show premature atherosclerosis ^8^. In clear contrast, hypopituitary patients with more classic forms of AGHD usually have combined pituitary deficiencies, are insulin-resistant, and present several risk factors for vascular complications ^2^. These observations clearly demonstrate the heterogeneous nature of AGHD and highlight that the findings in one specific group may not necessarily be applicable to other groups of patients with AGHD.

Whether GH therapy is able to normalize or decrease mortality rates in hypopituitary patients is still an unsolved question. A nationwide registry study from Denmark found that in GH-treated patients, total mortality, and mortality due to malignancy were decreased compared with untreated patients even after adjustment for all possible measured confounders ^9^. Data from the Dutch National Registry of GH treatment in adults found gender differences in the effects of GH treatment on mortality. AGHD men receiving GH treatment had a mortality rate not different from that of the background population, while the standardized mortality ratio (SMR) due to cardiovascular disease was still increased in women even after exclusion of high-risk patients ^10^. A study from the KIMS database (Pfizer International Metabolic Database) involving 13, 983 GH-replaced hypopituitary patients found a modest increase in all-cause mortality (SMR 1.13, 95% confidence interval 1.04–1.24), lower than values previously published in untreated AGHD ^11^. Factors associated with increased mortality in the KIMS database were female gender, younger attained age, aggressive pituitary tumors, and lower insulin-like growth factor 1 (IGF-I) standard deviation score during therapy. Of note, cardiovascular disease and malignancy were the leading causes of death, but the overall SMR for these cause categories was not significantly increased ^11^. Accordingly, in another epidemiological study from the Swedish KIMS database, in which 99.7% of hypopituitary patients were receiving GH treatment, excess mortality was associated not with vascular diseases but with two preventable causes of death: adrenal crisis in response to acute stress and intercurrent illness, and increased risk of a late appearance of *de novo* malignant brain tumors in patients who previously received radiotherapy. The observed shift in the causes of death were claimed to be related not only with GH therapy but with improvements in surgical procedures and hormone replacement regimens over the last 10 to 15 years, less use of radiotherapy, and more efficient therapies to manage cardiovascular risk factors ^12^.

AGHD should not be confused with states of functional GHD, such as obesity and aging. Aging is associated with a physiological decline in GH and IGF-I secretion, sometimes referred to as “somatopause” ^13– 19^. It is important to emphasize that neither the safety nor the benefits of GH administration to re-establishing “youthful” GH levels in pituitary-replete adults have been demonstrated to justify the use of GH as an anti-aging agent, and in most countries it is illegal to prescribe GH for off-label indications ^17– 20^. Having these concepts in mind, this review will focus on who the adult candidates for GH therapy are, the criteria for a biochemical diagnosis of severe AGHD, and the optimal therapeutic approach and the impact of GH therapy in AGHD, including efficacy and safety issues. In the last part, a brief overview of the long-term GH preparations will be presented.

## Adult candidates for growth hormone therapy

**Figure 1.**
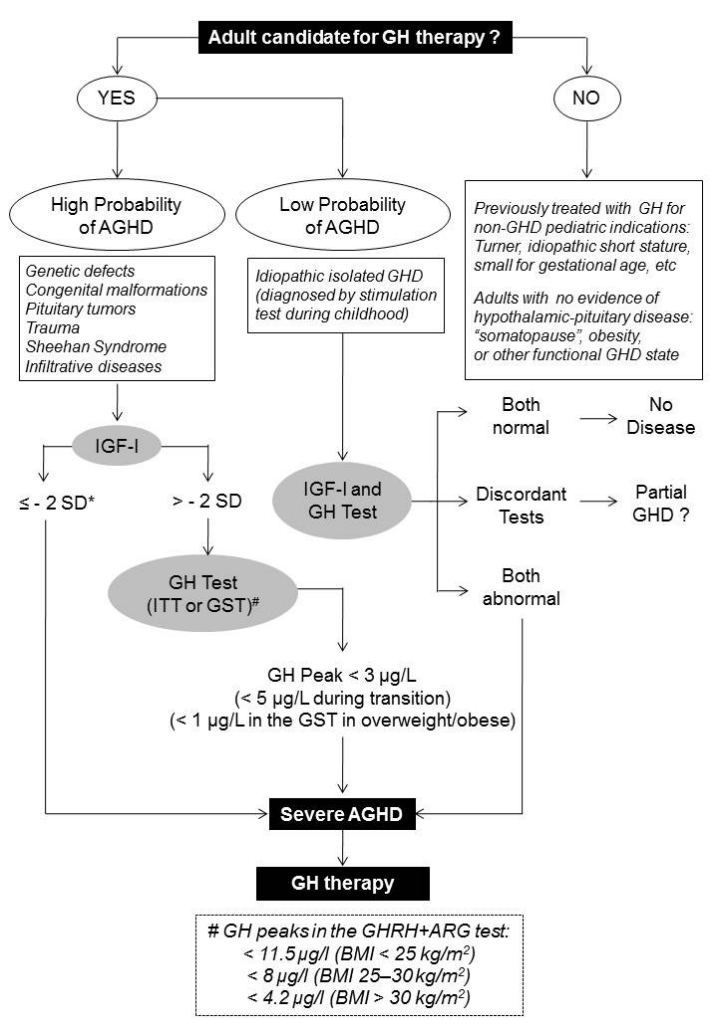
Diagnostic approach of adult growth hormone deficiency (AGHD). Asterisk indicates that “– 2 SD (standard deviation)” corresponds to IGF-I levels below the lower limit of the reference values adjusted for age. Hash sign indicates that, if available, growth hormone–releasing hormone plus arginine (GHRH+ARG) test is the best alternative for ITT, and corresponding diagnostic peak values are shown in the dashed rectangle. BMI, body mass index; GH, growth hormone; GHD, growth hormone deficiency; GST, glucagon stimulation test; IGF-I, insulin-like growth factor 1; ITT, insulin tolerance test.

AGHD should be investigated in all patients with evidence of a disease or trauma affecting the hypothalamic-pituitary region and in whom there is an intention to start treatment with GH ^13– 16^. This concept is pivotal, as adults with no well-established hypothalamic-pituitary disease, in whom GH and IGF-I levels might be functionally reduced because of aging or excess of visceral adipose tissue, are not candidates for GH therapy and therefore must not be subjected to diagnostic procedures ^13– 16^. The same concept is valid for short children treated with GH during childhood for non-GHD pediatric indications, such as Turner syndrome, small for gestational age, and idiopathic short stature ( Figure 1).

The causes of AGHD are multiple and include genetic defects; congenital malformations; trauma; sub-arachnoid hemorrhage; non-functioning and functioning pituitary adenomas (for example, prolactinoma, Cushing’s disease, and iatrogenic consequence of the acromegaly treatment); craniopharyngiomas; infiltrative, inflammatory, and vascular diseases; neurosurgery; and radiotherapy ^13– 16^. Traditionally, pituitary adenomas or their treatment (or both) have been listed as the most frequent causes of AGHD, but more recent studies have demonstrated an increasing prevalence of non-tumoral disorders, including traumatic brain injury and head trauma related to sports (boxing, kickboxing, soccer, football, ice hockey, and rugby) ^21^. Additionally, some studies have highlighted the importance of Sheehan’s syndrome as one of the leading causes of hypopituitarism and AGHD in adult women living in underdeveloped and developing countries ^21^. Manifestations of AGHD, owing to its heterogeneous nature, are variable, depending on how and when the disease is installed. Thus, AGHD can be diagnosed in association with other pituitary hormone deficiencies (combined AGHD) or alone (isolated AGHD). The disease can begin *de novo* in adult life (adulthood-onset) or can be diagnosed by GH tests in childhood persisting into adult life (childhood-onset) ^22^. In our hands, 56% of patients with idiopathic isolated GHD during childhood had no biochemical criteria for AGHD when re-tested in adult life, whereas 94% of those with combined GHD during childhood persisted with AGHD ^23^. Finally, another pivotal concept is related to the severity of the disease. Only patients with severe AGHD (as defined by strict biochemical criteria) are appropriate candidates for GH replacement therapy, as partial AGHD (or GH insufficiency) is still a poorly defined entity in which GH therapy is not indicated ^24^.

## Biochemical diagnosis of adult growth hormone deficiency

The clinical findings of AGHD are non-specific and usually of little diagnostic value ( Table 1). Thus, the presence of AGHD can be confirmed only by using an appropriate biochemical approach with GH and IGF-I measurements. The insulin tolerance test (ITT) and the GHRH plus arginine (GHRH+ARG) stimulation test have been traditionally used as the choice diagnostic procedures for AGHD ^2, 13– 16^. However, the GHRH analogue is not widely available, making the GHRH+ARG test impractical in many countries around the world ^25^. As a consequence, the glucagon stimulation test (GST) has emerged as the best alternative for cases in which ITT is unsuitable or contraindicated ^25– 29^. Some provocative tests frequently used to investigate GHD in children, which include arginine, clonidine, and L-dopa, are not valid for diagnosing AGHD ^13– 16^. Each GH stimulation test has advantages and limitations, and the choice must take in account contraindications, risks, and availability. It is also important to consider the high variability of GH results produced by different GH assays and to be aware of the assay and laboratory used for GH measurements ^30^. In most cases, an impaired peak GH level in one test is sufficient to diagnose AGHD ^13– 16^.

**Table 1.** Clinical abnormalities in hypopituitary adult patients with growth hormone deficiency (AGHD).

Clinical abnormalities	Effect of growth hormone deficiency
Changes in body composition	Increased total and visceral adiposity Reduced body lean mass Reduced bone mineral density (especially in transition and young adults)
Impairment of cognitive and psycho-social functions (poor quality of life)	Fatigue (low energy, reduced vitality) Low self-esteem Bad mood Reduced concentration Reduced memory Increased sick days Greater social isolation
Altered physical capacity	Reduced muscular strength Reduced maximum oxygen uptake Impaired cardiac function Hypohydrosis
Presence of cardiovascular risk factors	Dyslipidemia Insulin resistance (visceral obesity) Abnormal fibrinolytic activity Increased pro-inflammatory markers Increased intimal media thickening

Peak GH values in GH tests are influenced by several factors, including age, adiposity, and the type of GH secretagogue ^31– 33^. In the ITT, the validated cutoff value for biochemical diagnosis of severe AGHD is a peak GH response lower than 3 µg/L ^13– 16^. Nevertheless, the threshold value differs in patients in the transition phase—a period of time arbitrarily defined as “a 6–7 years interval spanning from late puberty (after achievement of final height) to the full adult somatic maturation” ^12, 29^. In the transition, a higher cutoff of 5 µg/L ^34^ or 6.1 µg/L ^35^ in the ITT has been recommended for the biochemical diagnosis. The cutoff value of 3 µg/L has also been recommended to discriminate between normal response and severe AGHD in the GST ^13– 16, 26^, but a lower threshold of 1 µg/L was recently proposed for overweight/obese subjects, as obesity blunts GH response to glucagon stimulation ^28, 36^. Similarly, cut-point levels in the GHRH+ARG test should be interpreted according to body mass index (BMI): 11.5 µg/L for patients with a BMI of less than 25 kg/m ^2^, 8 µg/L for a BMI of 25–30 kg/m ^2^, and 4.2 µg/L for those with a BMI of greater than 30 kg/m ^2^ ^32^.

GH stimulation tests are not always necessary for the final diagnosis of AGHD. As shown in Figure 1, the investigation can be oriented by the pre-test probability for AGHD. When there is a high probability of AGHD, single IGF-I measurements might be sufficient to make the diagnosis. For instance, in hypopituitary patients with three or more pituitary deficiencies, especially those who are younger than 40 years and have a BMI of below 25 kg/m ^2^, a low serum IGF-I level (below the lower limit of the reference range for age), in the absence of catabolic conditions or liver diseases, is highly indicative of severe AGHD ^13– 16^. In contrast, a normal serum IGF-I level in a hypopituitary patient does not exclude AGHD at any age, and the investigation should continue with a GH test. One important point to be highlighted is that the interpretation of IGF-I measurements can be challenged, as the results are highly variable among different assays and influenced by several factors, such as assay characteristics, gender, age, and reference values ^13– 16, 30^. In cases in which the pre-test probability of AGHD is low, as in transition patients diagnosed with idiopathic isolated GHD by stimulation tests during childhood or in obese and elderly hypopituitary patients, both GH test and IGF-I measurements are needed for establishing the diagnosis ^15, 34^.

## Impact of growth hormone therapy in adult growth hormone deficiency

GH replacement therapy might normalize abnormalities related to the AGHD state and promote numerous health benefits for hypopituitary patients ^2, 13– 16, 19, 20, 37^. In young adults with persistent GHD in the transition period, continuation or reintroduction of replacement treatment with GH after attaining final height promotes full somatic development of bone and muscles and prevents the development of AGHD features observed in older patients ^15, 34, 35, 38, 39^. GH treatment in AGHD reduces total and visceral fat mass, increases lean body mass and bone mass, and improves exercise capacity and quality of life (QoL) ^13– 16^. A meta-analysis of placebo-controlled trials showed an improvement in cardiovascular risk markers, including diastolic blood pressure, total cholesterol, and low-density lipoprotein cholesterol ^40^. Another meta-analysis found strong evidence that GH replacement improves exercise performance in AGHD ^41^. Recently, two meta-analyses have suggested a beneficial effect of GH therapy on bone mineral density in AGHD, which was mainly affected by gender, age, GH dose, and treatment duration ^42, 43^. In relation to QoL, one meta-analysis showed a significant difference in favor of GH therapy only on the Nottingham Health Profile social isolation dimension ^44^, but in cohorts of AGHD followed up to 10 years, a sustained improvement in QoL scores toward normality has been demonstrated, and there were more marked effects in women and in patients with low QoL at baseline ^45, 46^. It is worth mentioning that analysis of pros and cons of GH therapy might suffer interference from the associated pituitary deficiencies and from the adequacy of other replacement therapies. For instance, the avoidance of over- or under-replacement with glucocorticoids, sex steroids, and levothyroxine is fundamental to improve morbidities that are frequently associated with hypopituitarism ^47, 48^.

The clinical response to GH therapy is highly variable among patients with AGHD and is influenced by gender, age, BMI, GH dose, and the route of estrogen replacement in hypopituitary women ^2, 13– 16, 49, 50^. In a collaborative study between my institution and the Sahlgrenska Academy, University of Gothenburg, Sweden, we have developed mathematical models based on clinical parameters aimed at identifying good and poor responders to GH therapy ^51^. Using 1-year changes in serum IGF-I and body composition as therapeutic outcomes, we found that gender, body height, lean body mass, and serum insulin levels were the main clinical predictors of GH response in our models. In addition, we have investigated genetic factors that could be implicated with the variable response to GH therapy at different endpoints ^52– 54^. Using a candidate gene approach, we could identify associations between individual polymorphisms in genes related to lipid metabolism with serum lipid profile and in genes related to renal tubular function with extracellular water volume and blood pressure in AGHD patients both at baseline and after 1 year of GH therapy ^53, 54^. In two other studies, we examined the impact of the exon 3-deleted GH receptor (d3-GHR) isoform on the short-term (1 week) and long-term (1 year) responses to GH therapy in AGHD ^51, 55^. We found that carriers of the d3-GHR isoform had a poorer IGF-I response to GH after 1 week of therapy than full-length GHR homozygote individuals but that such an association was not observed after 1 year. This discrepant result might be explained by the fact that long-term studies are more subject to confounders such as dose titration and adherence to the treatment than the short-term model. The GHR polymorphism has been studied in other cohorts of patients with AGHD and the results, as expected, have been conflicting ^56– 62^. Thus, it is not clear at this moment whether the GHR polymorphism exerts some influence on GH responsiveness in adults, but if it does, the magnitude of the effect is likely to be modest. Further prospective, multicenter, pharmacogenetics studies in larger populations of patients with AGHD might help to define the relevance of clinical and genetic factors for GH responses, allowing the development of good prediction models to optimize therapeutic outcomes.

GH replacement is, in general, a safe and well-tolerated therapy in hypopituitary patients. However, special attention should be given to older, heavier, and female patients with AGHD because they are more susceptible to adverse events ^13– 16^. Individuals with AGHD are insulin-resistant because of increased adiposity, reduced lean body mass, and impaired physical performance. Insulin resistance might temporarily worsen in the first months of treatment because of diabetogenic effects of GH ^63^. The latter is usually compensated in the long-term therapy by the lipolytic actions of GH. Nevertheless, close monitoring of glucose status is advisable during GH treatment in a subset of AGHD patients with obesity or a family history of type 2 diabetes or both, because they are more prone to exhibit impaired glucose tolerance and diabetes mellitus in the first year of therapy ^20^. Owing to GH effects on thyroid hormones and cortisol metabolism, a pre-existing subclinical central hypothyroidism and secondary adrenal insufficiency might be unmasked during GH therapy. Therefore, patients should be carefully monitored for clinical manifestations of adrenal insufficiency or hypothyroidism, and free thyroxine and cortisol levels should be followed regularly during the initial phases of the treatment ^13– 16, 20, 64^.

Despite the potential role of GH and IGF-I in cell proliferation ^65^, there is no evidence of increased recurrence rates of intra-cranial or extra-cranial tumors among patients taking GH for AGHD ^13– 16, 20, 66^. However, GH should not be given to patients with active malignancy ^13– 16, 20^. There are few reports of long-term studies quantifying the increased risk, if any, of *de novo* malignancies. A recent Swedish study involving 426 patients with non-functioning pituitary adenomas—of whom 207 received GH therapy for a median period of 12.2 years and 219 were untreated patients with a median follow-up of 8.2 years—showed a reduced overall mortality in GH-treated patients compared with the general population and found that death due to malignancy was not increased in GH-treated patients ^67^. In addition, a recent meta-analysis including two retrospective and seven prospective studies with a total of 11,191 participants presented a reassuring conclusion that GH replacement therapy was associated with a reduced risk of cancer in AGHD ^68^. Nevertheless, it appears to be a slight excess risk of second neoplasia related to GH therapy among “high-risk” children, including patients with syndromes, diseases, and mutations known to be associated with an inherent elevated risk for cancer and early mortality, such as those with neurofibromatosis type 1, Fanconi anemia, and Down syndrome, but no comparable data are available for patients with AGHD ^20^. Hence, additional caution should be taken in AGHD patients with a history of cancer, strong family history of cancer, and advancing age ^66, 69^. Taken together, the evidence available at present demonstrates that the benefits of GH therapy in AGHD outweigh the theoretical cancer risk and that the screening for malignancies in GH-treated adults should follow the same rules applied for the general population ^20, 66^.

## Therapeutic approach in adult growth hormone deficiency

**Figure 2.**
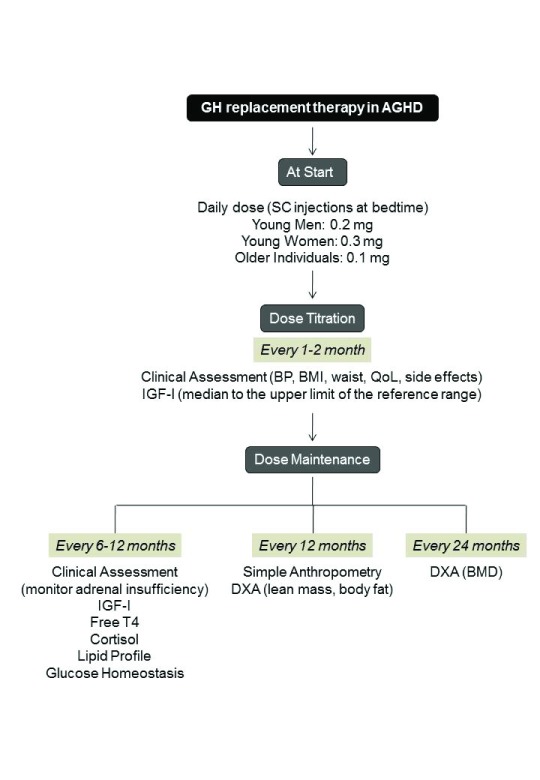
Therapeutic and follow-up procedures for growth hormone (GH) replacement therapy in adult growth hormone deficiency (AGHD). BMD, bone mineral density; BMI, body mass index; BP, blood pressure; DXA, dual-energy x-ray absorptiometry; IGF-I, insulin-like growth factor 1; QoL, quality of life; SC, subcutaneous.

In contrast with GH treatment in children, the replacement doses of GH in AGHD are not calculated on the basis of body weight, because this approach results in a high frequency of adverse events, mainly related to fluid retention: paresthesias, joint stiffness, peripheral edema, arthralgia, myalgia, and carpal tunnel syndrome ^2^. Thus, GH therapy in adults is initiated with low doses, which are progressively up-titrated to attain normal IGF-I levels ^13– 16^. At the start, the usual recommendation is a daily dose of 0.2 mg for young men, 0.3 mg for young women, and 0.1 mg for older individuals, which should be subcutaneously administered at bedtime ( Figure 2). In patients transitioning from pediatric treatment, initial doses typically have been intermediate between those used in the pediatric and in the adult population, which can be as high as 0.5–0.7 mg ^13, 14, 70^. The dose of GH is then increased gradually, at intervals of 1 to 2 months, in an individualized manner and guided by clinical assessments, side effects, and serum IGF-I levels. The objective is to attain and maintain normal IGF-I levels, preferably between the median and upper limit of the age-related normal range, always using the same IGF-I assay during the follow-up ^13– 16, 30^. There are scarce data specifically addressing how to monitor therapy in AGHD patients with normal IGF-I levels at baseline ^70^. As a general rule, however, the same dose titration approach should be applied with the aim of increasing IGF-I levels into the upper normal range and simultaneously monitoring efficacy through other therapeutic outcomes, such as body composition, cardiovascular risk factors, and QoL. Despite the importance of attaining normal IGF-I levels, in one of our previous studies we observed a significant impact of GH therapy on body composition with a fixed low-dose regimen maintained during 1 year, even in cases where IGF-I levels were not normalized ^71^. On the other hand, IGF-I levels above the upper limit of normal during GH therapy should always be interpreted as over-treatment, and dose adjustment is mandatory ^2, 13– 16^.

Once the maintenance dose of GH is achieved, IGF-I levels should be measured every 6 or 12 months to ensure that levels are kept in the normal range and below the upper limit to avoid over-treatment. As previously mentioned, the maintenance dose varies considerably from patient to patient, depending on factors such as age, gender, adiposity, and hormone interactions ^37– 39, 48– 50, 64^. For example, oral estrogen has been shown to have GH-antagonist effects; consequently, hypopituitary women in use of oral estrogen need much higher doses of GH to normalize IGF-I levels ^50, 64^. Therefore, as soon as GH treatment is prescribed, a non-oral route of exogenous estrogen should be prescribed for hypopituitary women ^13– 16, 50, 64^. Similarly, levothyroxine therapy might require adjustments, as weight-based doses of thyroid hormone are usually higher in AGHD patients under GH treatment ^13, 72, 73^. Conceptually, GH replacement should be a lifelong treatment, but its length has been a matter of debate. The physiological decline of GH secretion during the aging process is one reason for those who advocate stopping the treatment as patients get older. In contrast, there have been claims that there is no reason to stop treatment if clear benefits are being persistently achieved. Notably, regardless of the patient’s age, if no apparent or objective benefits of treatment have been observed after at least 1 year of follow-up, the discontinuation of GH therapy should be considered ^13, 37^.

Disease-generated questionnaires such as “Assessment of GHD in Adults” (QoL-AGHD) and general questionnaires such as “Psychological General Well-Being” and “Nottingham Health Profile” have been validated to measure a variety of health-related, economic, and social factors and are mainly used as research tools ^74, 75^. In some specialized centers, however, QoL-AGHD has been applied as a key tool for both starting and monitoring GH therapy in patients with AGHD. In most clinical settings, where these questionnaires are unavailable, health-related QoL outcomes, such as energy levels, partner satisfaction, sick days, and vitality, can be monitored by careful anamnesis before and after GH therapy, allowing a comprehensive view of potential therapeutic benefits in these parameters ^13– 16^. In untreated AGHD, QoL evaluations have shown a high degree of variability, from normal to severe impairment, affecting mainly energy and vitality. As a rule, if the QoL is normal at baseline, no major improvement should be expected with GH therapy, while the degree of improvement is generally proportional to the deviation from normality ^13, 45, 46^.

During GH therapy, changes in body composition need to be documented. While simple anthropometry with determination of BMI, skin folds, and waist circumference are easy to record, more objective measurements are preferable. Ideally, patients should have pre-treatment dual-energy x-ray absorptiometry, which should be repeated every one or two years to assess fat mass and lean body mass changes and every two years to evaluate bone mineral density changes. It is recommended that cardiovascular risk factors (particularly blood pressure, lipid profile, and glucose) be evaluated every 6 or 12 months ^13– 16, 37^.

## Future perspectives

Daily subcutaneous injections of GH might be laborious and cumbersome for some patients, leading to poor adherence that increases over time and compromises therapeutic efficacy. In this context, long-acting GH (LAGH) preparations requiring one injection per week, every two weeks, or once a month may offer a possible alternative to daily subcutaneous injections. As summarized in Table 2, there are LAGH preparations in various stages of development, including depot, pegylated, and prodrug formulations as well as non-covalent albumin-binding GH and GH-fusion proteins compounds ^76^. A review of efficacy and safety of LAGH preparations was recently published after an expert workshop held by the Growth Hormone Research Society ^77^. The expert panel concluded that each LAGH formulation has different pharmacokinetics and pharmacodynamics and therefore safety issues need to be addressed in an individualized and specific manner, with no comparison with those related to daily GH injections. Among the main safety concerns are the maintenance of supra-physiological elevations of GH or IGF-I levels (or both) and non-physiological tissue distribution ^76, 77^. As long-term data of LAGH preparations are unavailable, only prolonged monitoring will help to shed light on their safety profiles.

**Table 2.** Overview of the currently available long-acting growth hormone preparations.

Formulation	Product	Structure	Frequency of administration	Current status
Depot	LB03002	Microparticles containing GH incorporated into sodium hyaluronate and dispersed in an oil base of medium-chain triglycerides	Every week	Approved in Europe Approved in Korea for children with GHD
Pegylated	CP016	Nasal spray formulation	Every 2 weeks (planned)	Preclinical studies
Pegylated	BBT-031	Site-specific pegylated GH analog	Every week (planned)	Preclinical studies
Pegylated	Jintrolong	40-kDa polyethylene glycol attached to GH	Every week	Approved in China only for children with GHD
Prodrug	TransCon (ACP-001)	Unmodified GH transiently linked to a carrier molecule	Every week	Phase 2 studies in children and adults
Prodrug	NNC0195-0092	Single-point mutation in GH molecule with albumin binding moiety attached	Every week	Phase 2 studies in children and phase 3 in adults
GH fusion protein	ProFuse GH	GH-binding protein	Every month (planned)	Preclinical studies
GH fusion protein	GX-H9	Hybridization of non-cytolytic immunoglobulin Fc portion of IgD and IgG4	Every week or two weeks	Phase 2 studies in adults
GH fusion protein	LAPSrhGH/HM10560A	Homodimeric aglycosylated IgG4 Fc fragment	Every week or two weeks	Phase 2 studies in adults
GH fusion protein	MOD-4023	Carboxyl-terminal peptide of hCG beta- subunit	Every week	Phase 2 studies in children and phase 3 in adults
GH fusion protein	VRS-317	rhGH plus long chains of natural hydrophilic amino acids (XTEN sequence)	Every week or two weeks	Phase 3 studies in children and phase 2 in adults

GH, growth hormone; GHD, growth hormone deficiency.

The LAGH used in clinical trials thus far did not result in tachyphylaxis and showed similar clinical effects in comparison with daily GH injections ^76, 77^. Hoffman *et al*. ^78^ carried out a multicenter, open-label, randomized trial including 135 patients with AGHD: 51 received a depot GH preparation every two weeks, 53 received daily GH injections, and 31 were not treated. After 32 weeks, GH-treated groups showed a significant reduction of trunk and visceral adipose tissue and an increase in lean body mass in comparison with the untreated group. The depot formulation was as effective as daily GH injections. Similarly, in a placebo-controlled trial, Biller *et al*. ^79^ observed a significant reduction of fat mass (mainly due to decreased trunk fat) and increase in lean body mass in AGHD patients after 6 months of treatment with a sustained-release GH formulation. This trial continued in an open-label extension phase up to 52 weeks, which confirmed a prolonged and sustained effect of the LAGH preparation in reducing fat mass and increasing lean body mass, serum IGF-I, and IGF-binding protein 3 (IGFBP-3) level ^80^. While these preliminary data reassure the non-inferiority of LAGH preparations in relation to daily GH injections, their efficacy and safety over years and their implication for costs and compliance remain to be elucidated in ongoing and future clinical trials.

## Closing remarks

This review has tried to offer a pragmatic approach to the management of AGHD. Currently, adult patients with evidence of any abnormality of the hypothalamic-pituitary area should be considered for evaluation of severe GHD and replacement therapy with GH. AGHD is a heterogeneous condition characterized by multiple, non-specific, clinical abnormalities whose diagnosis is based on GH tests and single IGF-I measurements. The ITT and GHRH+ARG are preferable biochemical tools, and the GST is the best alternative for cases in which these tests are unavailable or contraindicated. In cases in which the probability of AGHD is very high, a low IGF-I level is sufficient for diagnosis, without the need for additional provocative testing. In cases in which the probability is low, a normal IGF-I level does not exclude the diagnosis and performing a GH test is imperative. The question of whether GH therapy reduces mortality in hypopituitarism has no definite answer yet. What is well recognized is that GH therapy can correct or improve clinical abnormalities seen in patients with AGHD, resulting in beneficial effects on body composition, skeletal integrity, exercise capacity, QoL, and cardiovascular risk factors. Protocols for GH dosing regimens and guidelines for monitoring efficacy and safety are well established and easily available. Observational studies with at least 7 years of follow-up have demonstrated that many clinical benefits of GH therapy in patients with AGHD are sustained in the long term with a low prevalence of serious adverse events and complications ^46, 81– 86^. More research is needed in some key areas, such as better characterization of partial AGHD, optimization of care during transition, development of mathematical models to predict individual responses to GH therapy, impact of GH therapy in the mortality rates of hypopituitary patients, and therapeutic potential of the LAGH preparations.
